# Assessing the Efficiency and Productivity of the Hospital Clinics on the Island of Rhodes during the COVID-19 Pandemic

**DOI:** 10.3390/ijerph192315640

**Published:** 2022-11-24

**Authors:** Lorena Androutsou, Michail Kokkinos, Dimitra Latsou, Mary Geitona

**Affiliations:** 1 Department of Economics and Business, School of Economics, Business and Computer Sciences, Neapolis University Pafos, Pafos 8042, Cyprus; 2 Ophthalmology Department, General Hospital of Rhodes, 85100 Rhodes, Greece; 3 Department of Social and Educational Policy, School of Social Sciences, University of Peloponnese, 20132 Corinth, Greece

**Keywords:** efficiency, productivity, data envelopment analysis (DEA), Malmquist, hospital, COVID-19, Greece

## Abstract

(1) Background: The aim was to measure the efficiency and productivity of 15 specialty clinics during the COVID-19 pandemic period 2020–2021 in the General Hospital of Rhodes. (2) Methods: An input-oriented data envelopment analysis and the Malmquist productivity index are used. Labor and capital were used as inputs, and in-patient discharges and days were used as outputs. (3) Results: Five out of the seven clinics in the pathology sector appeared fully efficient with an optimal productivity, and the rest showed progress in 2021. In 2020 the COVID-19 pathology clinic appeared to be inefficient and less productive, while in 2021, it showed a positive performance change. The surgical sector showed very high efficiency rates or even reached an optimal efficiency in both years. The productivity measurement, in most of the surgical clinics, was satisfactory to very high. In 2020 the COVID-19 surgical clinic appeared to be more efficient and productive than in 2021 when its performance declined. (4) Conclusions: The hospital responded to the pressure during the pandemic, by increasing its efficiency and productivity from 2020 to 2021. This was due to the accomplishment of the appropriate organizational changes in the infrastructure, human resources, and technology. The efficiency and productivity assessments should be incorporated in the hospitals’ decision making.

## 1. Introduction

The COVID-19 health crisis intensified the cooperation and solidarity between states and organizations, boosted technological advances, and redefined new pathways in resources’ management. More importantly it emphasized accessibility, sustainability, and created opportunities and new perspectives in health care systems [1,2]. This situation resulted to an increased health care expenditure [3], urged the necessity to adopt control systems, and apply effectiveness and efficiency criteria in health systems [4]. Hospitals had to develop new clinical, managerial, and public health practices focusing on COVID-19 care, while simultaneously, they had to sustain the efficiency and quality of healthcare. Thus, the need for better resources’ management and performance assessments focusing on the measurement and comparative analysis of efficiency and productivity, became more demanding, in order to highlight the best practices.

Efficiency and productivity are interrelated concepts [5]. Efficiency refers to the “comparison between an existing function with the optimal one” [6]. Productivity is the measure of the production capacity, including the measurement of efficiency to achieve the greatest performance with the lesser effort [7,8]. The data envelopment analysis (DEA) method is a key tool for performing these measurements. Charnes, Cooper, and Rhodes started, in 1978, the theoretical development of the DEA approach on how to produce a measure of efficiency for decision-making units (DMUs) [9]. This is a non-parametric method of mathematical programming capable of handling multiple inputs and outputs expressed in different measurement units, therefore it is considered as a multi-criteria approach. The units (organizations) that convert the inputs into outputs are referred to as DMUs and determine the best organizations (best-practice) to determine the “efficiency limit” [10]. The DEA provides two options while creating models: input or output oriented. The computation of DEA can be performed under both variable returns to scale (VRS) and the constant returns to scale (CRS). The CRS score captures the global (in)efficiency, whereas the VRS and the “scale” scores decompose the global score into pure technical efficiency and scale efficiency, respectively [9,11,12]. The conversion of the inputs into outputs relative to best practice is referred to as the technical efficiency. Depending on the input-output ratio of efficiency, it provides the ability of an organization to produce as much output as the input allows, or to use as little input as the output production allows [9,13].

The DEA also includes the Malmquist productivity index (MI) for each DMU. Founded by Malmquist [14], adopted by Caves et al. [15], and developed by Färe et al. [12] as a productivity indicator, based on the DEA, it measures the productivity change, diachronically. The VRS/CRS option has no influence on the Malmquist DEA because both are used to calculate various distances used to construct the Malmquist indices [16,17]. The application of this non-parametric mathematical programming method is a commonly used technique for recording data and calculating indicators of the total productivity change and its components (technological change, pure technical efficiency, scale efficiency) [5,6,9,13,15,16,17,18,19,20]. The DEA analysis has been widely used in other economic sectors and the theoretical modeling has been extended with the combination of new variables [9,12,15,19,21,22,23,24,25,26,27,28,29].

The number of studies, based on the DEA methods, is gradually increasing and the DEA is the most frequently used tool for the efficiency evaluation [30], with healthcare and hospitals being among the most popular topics [31]. When applying the DEA in health care, the DMU is usually defined as an entire hospital or clinic (homogeneous or heterogeneous). The DEA facilitates a comparative analysis while evaluating the results for one year or diachronically. Studies in efficiency measurement, using parametric or non-parametric techniques in the hospital sector, have been conducted in several countries, including Greece [32,33,34,35,36,37,38,39,40,41,42,43,44,45,46,47,48,49]. However, the published bibliography assessing the efficiency at the clinical level by running the DEA [50,51,52], has been limited with only a single study examining the combined efficiency and productivity in Greece [42]. In early 2020, the COVID-19 pandemic was declared by the WHO, urging the need to reallocate resources and recruit personnel efficiently and productively, as one of the main measures to respond to this health system crisis.

Greece was also hit by the COVID-19 pandemic. The government responded immediately by introducing policies in the healthcare sector [53,54,55]. At the hospital level, numerous measures were implemented focusing on organizational, administrative, and managerial interventions. Fundamental changes included the reallocation of beds, personnel, and medical equipment, the clinics’ transformations into COVID-19 units, additional response funding, and patients’ management. Even though numerous studies have explored the efficiency and productivity of hospitals by using the DEA in Greece, there are no studies conducted during the COVID-19 pandemic. There is also a very limited number of similar studies worldwide.

Thus, the aim of this study was to assess the clinics’ efficiency and productivity of the General Hospital of Rhodes during the 2020–2021 pandemic, by applying the DEA and MI models. The hospital under study is located in the island of Rhodes, which is remote from the Greek mainland. On an annual basis, the hospital covers the patient care for approximately 350,000 people, including the residents of Rhodes and the neighboring smaller islands, as well as tourists and refugees [56]. During the examined years, the capacity of the hospital was 22 clinics, 400 beds, and 580 staff. More specifically, in 2020, there were 23,000 in-patients and approximately 60,000 hospitalization days, which increased slightly in 2021, to 24,000 and 65,000, accordingly. The COVID-19 patients were 405 in 2020 with 1200 hospitalization days, while in 2021, the patient number tripled to 1500 with a 525% increase in hospitalization days (7500).

## 2. Materials and Methods

A case study was conducted in the largest hospital of the South Aegean region. The methodology applied was the DEA and MI. The efficiency and productivity were assessed in heterogeneous and homogenous clinics. Out of the 22 clinics, seven were excluded from the analysis, based on the following criteria: intensive care units (ICUs) due to patients’ emergency profile feature, diagnostic examination unit, one day clinics, such as artificial kidney, Mediterranean anemia, infectious diseases, hemodynamic, and dental units. In addition, out-patient care was also excluded. In particular, 15 clinics were selected, seven in the pathology and eight in the surgical sector, that provided in-patient care. All 15 clinics were considered single DMUs. In order to create comparable units and to avoid the heterogeneity of the sample, two DEA models were built and separately run by sector, as presented in Table 1.

The scores for the MI measure the changes in the total factor productivity (TFPCH), technological and technical efficiency (TECHCH and EFFCH, respectively) and its components (pure technical efficiency change -PECH and scale efficiency change-SECH) [15]. More specifically, the technological efficiency change shows the extent to which the technological limit of the examined DMU is shifted from one period to another [57]. A score higher than one indicates productivity progress. When the technical efficiency exceeds the technological change index, then productivity growth is primarily the result of the technical efficiency improvements. When the opposite occurs, then the increased efficiency is mainly attributed to the technological progress [6,58,59]. Furthermore, the technical efficiency change affects the productivity through its components, the pure technical efficiency, and scale efficiency. Pure technical efficiency, which in turn expresses the application of the best practices of the resource usage and the scale efficiency, determines how closely an observed DMU is on the most productive scale [15,29]. Moreover, numerous studies have emphasized the decomposition of the productivity and the influential role of the technical and scale efficiencies in the hospital’s performance [60,61,62,63].

The DEA and MI methods chosen were input-oriented, run for the VRS and have been performed in clinics that were considered to transform labor and capital (inputs) into health services, approximated by the number of in-patient discharges and in-patient days (outputs). The annual number of full-time medical and nursing personnel employed in each clinic, is used as the proxy labor inputs. The number of hospital beds is assumed to be the proxy input of capital resources. The study uses a hospital clinic perspective.

The data were collected from the hospital’s medical records and through the bi-health system, which is the data registered annually and officially by the Regional Health Authority (2nd DYPE). The results were obtained using the software “DEA.P version 2.1 for Windows” [18]. The study was approved by the Scientific Council of the General Hospital of Rhodes.

## 3. Results

### 3.1. Analysis of Efficiency

**Figure 1:**
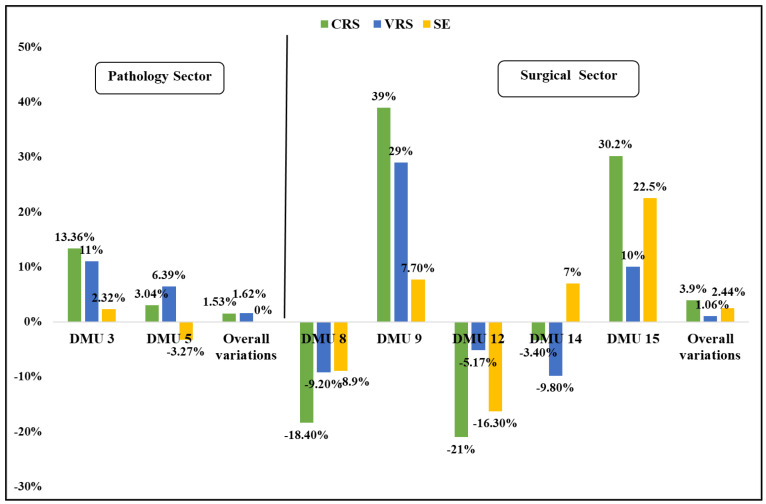
CRS, VRS, and SE variations (%) in the pathology and surgical sectors (2020–2021).

During the two years of the review (2020 and 2021), the pathology clinics (DMUs 1, 2, 4, 6, and 7) appeared fully efficient and approached an optimal efficiency (Table 2). In 2021, the A’ Pathology (DMU 3) was fully transformed into a COVID-19 clinic, by increasing its inputs (beds and nursing staff) and outputs (in-patient discharges and days), showing a very positive performance change, compared to 2020. This clinic became efficient in the CRS and VRS, as well in the SE, proving that the operational changes implemented have brought it very close to the most efficient performance, as shown in Figure 1. The pediatric clinic (DMU 5) showed a low efficiency under the CRS and VRS in 2020 and also remained at low levels in 2021 (Figure 1).

Regarding the surgical sector, very high-efficiency rates or even optimal rates were reached. In both 2020 and 2021, the surgical clinics (DMUs 10, 11, and 13) scored optimal efficiency levels under the CRS, VRS, and SE. The rest of the DMUs showed variations between the examined years, as shown in Table 2. More specifically, only DMU 9 and DMU 15 presented a significant improvement and demonstrated a full efficiency from 2020 to 2021 (Figure 1). DMUs 8, 12, and 14 showed a decrease in efficiency under the CRS and VRS, while the SE was differentiated among them (Figure 1).

Overall, the pathology sector was very efficient in 2020 and 2021, indicating positive variations in the second year of 1.53% and 1.62%, under the CRS and VRS, accordingly, and no noticeable variations in the SE. The surgical sector was also very efficient in both years, with positive variations in the CRS (3.9%), VRS (1.06%), and SE (2.44%) in 2021 (Figure 1).

### 3.2. Analysis of Productivity

**Figure 2:**
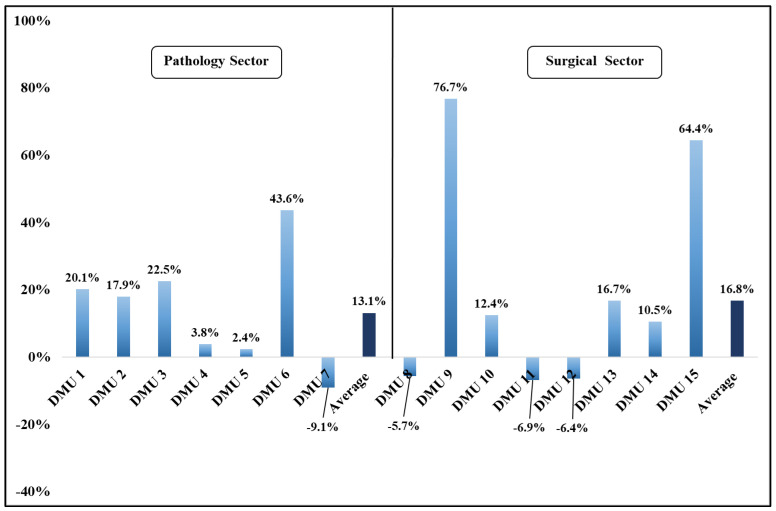
Total factor productivity change index (%) of the pathology and surgical sectors (2020–2021).

In 2021, all clinics of the pathology sector showed a positive change in the TFPCH, due to the significant increase in the technological change, except DMU 7, as presented in Table 3 and Table 4, and Figure 2. Moreover, in DMU 3 (the COVID-19 clinic), an increase in the TFPCH is observed, due to the positive changes in the EFFCH, PECH, SECH, and TECHCH, demonstrating a better use of inputs (personnel and beds) and reaching an optimal level of operation during 2021. Furthermore, in 2021, DMU 5 presented a positive change in the TFPCH counterbalancing the slight decrease in the TECHCH (−0.6%) and SECH (−3.3%). In DMU 7, a reduction of 9.1% in the TECHCH and TFPCH appeared, while the other indexes achieved higher than 1 threshold values.

Most of the clinics in the surgical sector showed a positive change in 2021, in the TFPCH (Table 3 and Table 4 and Figure 2). More specifically, the increase in the TECHCH of DMUs 10 and 13 impacted positively on the TFPCH. DMU 14 showed an increase in the TFPCH by 10.5%, which is due to the increase of the TECHCH and the SECH counterbalancing the decrease of the PECH by 9.8%. DMU 15 had a significant increase in the TFPCH by 64.4%, due to the improvement of the TECHCH and SECH. However, in DMUs 8, 11, and 12, the TFPCH was negatively affected by both the EFFCH and TECHCH. In particular, the negative TFPCH in DMUs 8 and 12 is explained, due to the decrease in the EFFCH, which is both at the level of the PECH and SECH, although the TECHCH increased. In DMU 11, despite the very productive performance, a reduction of 6.9% in the TECHCH impacted negatively the TFPCH. In addition, a significant variation was observed in the TFPCH between the homogeneous clinics, whereas DMU 9 presented an exceptionally positive change (76.7%), compared to a 5.7% negative change in DMU 8.

Overall, the TFPCH and all of the other indexes of the pathology and surgical sectors increased between 2020 and 2021, except for a slight reduction in the SECH of the pathology sector (0.2%) (Figure 2).

## 4. Discussion

The assessment of the hospital efficiency and productivity performances can be of great value at the micro and macro levels in the healthcare sector. The necessity for a comparative assessment becomes higher in a pandemic era, especially when healthcare needs and resource allocations increase and cannot be predicted. The aim of this study was to measure the efficiency and productivity of the hospital clinics in the General Hospital of Rhodes, during the 2020–2021 COVID-19 pandemic, by applying the DEA and MI models.

The results of our study showed that during both years in the pathology sector, five out of the seven clinics appeared fully efficient with an optimal productivity, while the other two showed progress in 2021. The A′ pathology clinic (DMU 3) was converted entirely into a COVID-19 unit in 2020, with a positive impact on the efficiency and productivity in 2021. This clinic not only increased the technological threshold but also improved the clinical practices by benefiting from the increase in human and capital resources, during the second period. The B′ pathology clinic (DMU 4), also operated at an optimal level by hospitalizing all of the non-COVID cases. Thus, both homogenous clinics achieved the best performance, verifying the soundness of the policies pursued.

Additionally, the surgical sector scored high-efficiency rates and satisfactory to high productivity rates. Three out of the eight clinics revealed optimal performance levels by fully exploiting their capital and human resources, as well as innovation, brought in the context of the pandemic to the hospital. The performance of the A’ surgical clinic (DMU 8) declined in 2021 because it was fully transformed into a COVID-19 unit. The efficiency and productivity of the B’ surgical clinic (DMU 9) were greatly increased, due to its excess capacity as the other homogenous clinic (DMU 8) operated as the “backup plan” for COVID-19 patients.

Regarding the changes in the total factor productivity in both sectors, it is observed that although the overall rates had a similar degree of increase, the technical efficiency affected more the clinics of the surgical sector. In addition, the shift in the technological change had a significant impact on the change in the total factor productivity in both sectors, with the pathological sector mostly affected.

It should be noted that the progress observed in the hospital clinics was due to a plethora of alternative organizational, administrative, and managerial interventions that were introduced during the pandemic outbreak. Among the most important measures taken were the introduction of medical innovations and equipment related to COVID-19 care, the transformation of specific clinics in both sectors to COVID-19 units, the reallocation of beds and staff, as well as the changing of patients’ management. Our results are in accordance with the international literature. There are numerous studies reporting that the processes related to the organization and management of health resources, the infrastructure, the availability, and use of innovative technologies, as well as patient management and staff training affect productivity changes [51,64,65,66,67]. Several studies have stated that productivity deficits are mostly due to the losses in operational capacity, limited planning ability, insufficient staff availability, and longer procedure implementation times [68,69,70,71]. Furthermore, the hospital structure and, in particular, healthcare equipment and human capital, play a crucial role in the management of the pandemic [72]. The Lytras and Tsiodras study conducted during the pandemic in Greece, highlighted not only the limited resources and the stress capacity depletion, but also the necessity for staff training and specialization [73]. Furthermore, the number of beds plays a significant role in the management of the pandemic along with the reallocation of patients and, subsequently the reallocation of beds [74]. Many authors also emphasize the effect of both clinic size and the number of nursing staff on both the technical efficiency and performance [65,66,68,71]. Another common finding is reported in a recent study conducted in Portugal during the pandemic showing that hospitals’ performance also dropped in 2020, while in 2021, better results were achieved [75]. Despite the fact that international research under pandemic conditions is limited, hospitals’ inefficiencies related to the diversity in their structure and management have been highlighted [74,76,77,78]. The loss of operational capacity, limited planning ability, insufficient staff availability, the inadequate response to non-COVID incidents and longer procedure implementation times, create a productivity deficit [70].

In Greece, many studies were conducted to measure the efficiency and productivity at the hospital and the clinical level by applying the DEA, with the majority of them being input oriented [32,33,36,39,40,43,44,45,47,48,49,50,79,80,81,82,83]. Only the studies of Androutsou, et al., in 2011 [42] and Geitona, et al., in 2013 [50] were output-oriented, comparing the homogeneous and heterogeneous hospital clinics. Moreover, several studies conducted before the pandemic in Greece reported inefficiencies in the hospitals’ clinics, mostly related to the lack of appropriate infrastructure, staff, the irrational utilization of financial and human resources, as well as innovation [32,33,36,39,42,49,50,79,80]. In some of the above-mentioned studies, it was also found that the pathological clinics were more efficient than the surgical ones [39,42,50].

### Limitations of the Study

Some limitations should be taken into consideration. First, the analysis is only based on a mathematical approach and is unable to examine the qualitative characteristics of the decision units. Second, the epidemiological data, readmissions rates, the severity of cases, as well as the specific clinics, based on the exclusion criteria, have not been taken into consideration. Third, our results come from a remote general hospital in Greece and cannot be generalized. However, they should not be underestimated, due to the fact that it is the first attempt to measure the efficiency and productivity of the hospital clinics under pandemic conditions in the country.

## 5. Conclusions

The General Hospital of Rhodes responded to the pressure created by the pandemic crisis by increasing its efficiency and productivity from 2020 to 2021. This achievement was due to its ability to accomplish the appropriate organizational changes and rationally allocate human resources, infrastructure, as well as technology. The efficiency and productivity assessments, especially during a pandemic crisis, should be considered as an opportunity to take advantage of the reorganization of hospitals, the clinics’ management and the integration of innovation. Moreover, successful measures taken can be a reference for best practices generated across the healthcare sector.

## Figures and Tables

**Table 1:** Clinics per sector.

Clinics of the Pathology Sector	Clinics of the Surgical Sector
DMU 1: Hematology	DMU 8: A’ Surgery-COVID-19
DMU 2: Cardiology	DMU 9: B’ Surgery
DMU 3: A’ Pathology–COVID-19	DMU 10: Obstetrics and Gynecology
DMU 4: B’ Pathology	DMU 11: Urology
DMU 5: Pediatric	DMU 12: Ear, Nose, Throat (E.N.T.)
DMU 6: Neurology	DMU 13: Ophthalmology
DMU 7: Gastroenterology	DMU 14: Orthopedics DMU 15: Neurosurgery
Total number of clinics: 7	Total number of clinics: 8

**Table 2:** Efficiency and productivity measurement of the pathology and surgical sectors (2020–2021).

	CRS		VRS		SE-CRS/VRS	
DMU	2020	2021	2020	2021	2020	2021
Pathology Sector						
DMU 1	1.000	1.000	1.000	1.000	1.000	1.000
DMU 2	1.000	1.000	1.000	1.000	1.000	1.000
DMU 3	0.651	0.738	0.686	0.761	0.948	0.970
DMU 4	1.000	1.000	1.000	1.000	1.000	1.000
DMU 5	0.296	0.305	0.360	0.383	0.824	0.797
DMU 6	1.000	1.000	1.000	1.000	1.000	1.000
DMU 7	1.000	1.000	1.000	1.000	1.000	1.000
Average	0.850	0.863	0.864	0.878	0.967	0.967
Surgical Sector						
DMU 8	0.934	0.762	0.982	0.879	0.951	0.867
DMU 9	0.720	1.000	0.775	1.000	0.929	1.000
DMU 10	1.000	1.000	1.000	1.000	1.000	1.000
DMU 11	1.000	1.000	1.000	1.000	1.000	1.000
DMU 12	0.552	0.436	0.848	0.800	0.651	0.545
DMU 13	1.000	1.000	1.000	1.000	1.000	1.000
DMU 14	0.912	0.881	1.000	0.902	0.912	0.976
DMU 15	0.698	1.000	0.901	1.000	0.775	1.000
Average	0.852	0.885	0.938	0.948	0.902	0.924

CRS = Constant Returns to Scale, VRS = Variable Returns to Scale, SE = Scale Efficiency.

**Table 3:** Malmquist productivity index of the pathology and surgical sectors (2020–2021).

DMU	EFFCH	TECHCH	PECH	SECH	TFPCH
Pathology Sector					
DMU 1	1.000	1.201	1.000	1.000	1.201
DMU 2	1.000	1.179	1.000	1.000	1.179
DMU 3	1.134	1.080	1.109	1.022	1.225
DMU 4	1.000	1.038	1.000	1.000	1.038
DMU 5	1.030	0.994	1.065	0.967	1.024
DMU 6	1.000	1.436	1.000	1.000	1.436
DMU 7	1.000	0.909	1.000	1.000	0.909
Average	1.022	1.109	1.024	0.998	1.134
Surgical Sector					
DMU 8	0.816	1.156	0.895	0.912	0.943
DMU 9	1.389	1.272	1.290	1.077	1.767
DMU 10	1.000	1.124	1.000	1.000	1.124
DMU 11	1.000	0.931	1.000	1.000	0.931
DMU 12	0.790	1.186	0.943	0.837	0.936
DMU 13	1.000	1.167	1.000	1.000	1.167
DMU 14	0.966	1.144	0.902	1.071	1.105
DMU 15	1.432	1.148	1.110	1.290	1.644
Average	1.023	1.137	1.011	1.016	1.168

EFFCH = technical efficiency change index, TECHCH = technological change index, PECH = pure technical efficiency change index, SECH = scale efficiency change index, TFPCH = total factor productivity change index.

**Table 4:** Malmquist productivity index changes (%) of the pathology and surgical sectors (2020–2021).

	EFFCH	TECHCH	PECH	SECH
Pathology Sector				
DMU 1		20.1%		
DMU 2		17.9%		
DMU 3	13.1%	8.0%	10.9%	2.2%
DMU 4		3.8%		
DMU 5	3.0%	−0.6%	6.5%	−3.3%
DMU 6		43.6%		
DMU 7		−9.1%		
Average	2.2%	10.9%	2.4%	−0.2%
Surgical Sector				
DMU 8	−18.4%	15.6%	−10.4%	−8.8%
DMU 9	38.3%	27.2%	29.0%	7.7%
DMU 10		12.4%		
DMU 11		−6.9%		
DMU 12	−21.0%	18.6%	−5.7%	−16.3%
DMU 13		16.7%		
DMU 14	−3.4%	14.4%	−9.8%	7.1%
DMU 15	43.2%	14.8%	11.0%	12.9%
Average	2.3%	13.7%	1.1%	1.6%

EFFCH = technical efficiency change index, TECHCH = technological change index, PECH = pure technical efficiency change index, SECH = scale efficiency change index.

## Data Availability

Restrictions apply to the availability of these data. Data were collected from the Post-Graduate Program “Public Administration-health services administration” of the Department of Economics and Business of the Neapolis University Pafos (NUP).
